# Triclinic form of bis­{di-μ-hydroxidobis[*fac*-aqua­tribromido­tin(IV)]} hepta­hydrate

**DOI:** 10.1107/S1600536810006021

**Published:** 2010-02-20

**Authors:** Geraldo M. de Lima, R. Alan Howie, Edward R. T. Tiekink, James L. Wardell, Solange M. S. V. Wardell

**Affiliations:** a Departamento de Quimica, ICEx, Universidade Federal de Minas Gerais, 31270-901 Belo Horizonte, MG, Brazil; b Department of Chemistry, University of Aberdeen, Old Aberdeen, AB15 5NY, Scotland; c Department of Chemistry, University of Malaya, 50603 Kuala Lumpur, Malaysia; d Centro de Desenvolvimento Tecnológico em Saúde (CDTS), Fundação Oswaldo Cruz (FIOCRUZ), Casa Amarela, Campus de Manguinhos, Av. Brasil 4365, 21040-900, Rio de Janeiro, RJ, Brazil; e CHEMSOL, 1 Harcourt Road, Aberdeen AB15 5NY, Scotland

## Abstract

The asymmetric unit of the title hydrate, 2[Sn(H_2_O)_2_(OH)_2_Br_6_]·7H_2_O, comprises two [Br_3_(H_2_O)Sn(μ-OH)_2_SnBr_3_(OH_2_)] units, but three independent mol­ecules as two of these are disposed about inversion centres, and seven water mol­ecules. In common with the monoclinic polymorph [Howie *et al.* (2005 ▶). *Inorg. Chim. Acta*, **358**, 3283–3286], each of the dinuclear species features a central Sn_2_O_2_ core, distorted octa­hedral Sn atom geometries defined by a Br_3_O_3_ donor set, and an *anti*-disposition of the coordinated water mol­ecules. In the crystal, O_h_—H⋯O_w_, O_a_—H⋯O_w_, O_w_—H⋯O_w_, and O_w_—H⋯Br (h = hydroxyl, a = aqua, w = water) hydrogen-bonding inter­actions generate a three-dimensional network.

## Related literature

For the structure of the monoclinic polymorph, see: Howie *et al.* (2005 ▶). For related di-μ-hydroxido-bis­[*fac*-trichlorido­aqua­tin(IV)] complexes, see: Barnes *et al.* (1980 ▶); Cameron *et al.* (1985 ▶); Shihada *et al.* (2004 ▶); Müller *et al.* (2007 ▶). For analysis of pseudo-symmetry, see: Spek (2003 ▶).
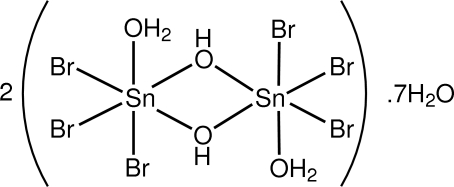


## Experimental

### 

#### Crystal data

[Sn_2_Br_6_(OH)_2_(H_2_O)_2_]_2_·7H_2_O
*M* *_r_* = 1699.85
Triclinic, 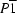

*a* = 9.9652 (2) Å
*b* = 14.0027 (3) Å
*c* = 14.5230 (3) Å
α = 64.8591 (13)°
β = 69.9803 (13)°
γ = 75.0492 (15)°
*V* = 1708.54 (6) Å^3^
*Z* = 2
Mo *K*α radiation
μ = 16.97 mm^−1^
*T* = 120 K
0.20 × 0.18 × 0.06 mm

#### Data collection

Nonius KappaCCD diffractometer
Absorption correction: multi-scan (*SADABS*; Sheldrick, 2007 ▶) *T*_min_ = 0.421, *T*_max_ = 0.746
36325 measured reflections
7823 independent reflections
5613 reflections with *I* > 2σ(*I*)
*R*_int_ = 0.045

#### Refinement

*R*[*F* ^2^ > 2σ(*F* ^2^)] = 0.035
*wR*(*F* ^2^) = 0.078
*S* = 1.05
7823 reflections
352 parameters
36 restraints
H-atom parameters constrained
Δρ_max_ = 1.06 e Å^−3^
Δρ_min_ = −1.63 e Å^−3^

### 

Data collection: *COLLECT* (Hooft, 1998 ▶); cell refinement: *DENZO* (Otwinowski & Minor, 1997 ▶) and *COLLECT*; data reduction: *DENZO* and *COLLECT*; program(s) used to solve structure: *SHELXS97* (Sheldrick, 2008 ▶); program(s) used to refine structure: *SHELXL97* (Sheldrick, 2008 ▶); molecular graphics: *ORTEP-3* (Farrugia, 1997 ▶) and *DIAMOND* (Brandenburg, 2006 ▶); software used to prepare material for publication: *publCIF* (Westrip, 2010 ▶).

## Supplementary Material

Crystal structure: contains datablocks general, I. DOI: 10.1107/S1600536810006021/hb5334sup1.cif

Structure factors: contains datablocks I. DOI: 10.1107/S1600536810006021/hb5334Isup2.hkl

Additional supplementary materials:  crystallographic information; 3D view; checkCIF report

## Figures and Tables

**Table:** 

Sn1—O3	2.078 (4)
Sn1—O2	2.082 (4)
Sn1—O1	2.140 (4)
Sn1—Br2	2.5078 (7)
Sn1—Br3	2.5100 (7)
Sn1—Br1	2.5830 (7)
Sn2—O2	2.070 (4)
Sn2—O3	2.082 (4)
Sn2—O4	2.176 (4)
Sn2—Br6	2.5062 (7)
Sn2—Br4	2.5180 (7)
Sn2—Br5	2.5726 (7)
Sn3—O6	2.080 (4)
Sn3—O6^i^	2.086 (4)
Sn3—O5	2.144 (4)
Sn3—Br9	2.5161 (6)
Sn3—Br7	2.5173 (7)
Sn3—Br8	2.5599 (7)
Sn4—O8	2.083 (4)
Sn4—O8^ii^	2.090 (4)
Sn4—O7	2.150 (4)
Sn4—Br11	2.5114 (7)
Sn4—Br12	2.5230 (6)
Sn4—Br10	2.5487 (7)

**Table:** 

Sn2—O2—Sn1	108.35 (16)
Sn1—O3—Sn2	108.06 (16)
Sn3—O6—Sn3^i^	108.54 (17)
Sn4—O8—Sn4^ii^	107.99 (17)

Symmetry codes: (i) 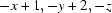; (ii) 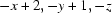.

**Table 2:** Hydrogen-bond geometry (Å, °)

*D*—H⋯*A*	*D*—H	H⋯*A*	*D*⋯*A*	*D*—H⋯*A*
O1—H1a⋯O15	0.84	1.75	2.573 (6)	165
O1—H1b⋯Br5	0.84	2.50	3.290 (4)	157
O2—H2⋯O11	0.84	1.80	2.638 (6)	172
O3—H3⋯O14^iii^	0.84	1.87	2.657 (6)	156
O4—H4a⋯O12	0.84	1.85	2.692 (6)	175
O4—H4b⋯Br1	0.84	2.51	3.257 (4)	149
O5—H5a⋯O10^iv^	0.84	1.76	2.592 (4)	174
O5—H5b⋯Br8^i^	0.84	2.60	3.329 (5)	146
O6—H6⋯O9^iv^	0.84	1.93	2.764 (7)	169
O7—H7a⋯O13	0.84	1.76	2.600 (6)	172
O7—H7b⋯Br10^ii^	0.84	2.64	3.307 (5)	137
O8—H8⋯O9^v^	0.84	1.99	2.768 (7)	154
O9—H9a⋯O12	0.84	1.98	2.817 (7)	175
O9—H9b⋯Br4	0.84	2.71	3.522 (4)	162
O10—H10a⋯Br1^vi^	0.84	2.87	3.456 (3)	129
O10—H10b⋯O11	0.84	2.14	2.772 (5)	132
O11—H11a⋯O13	0.84	1.97	2.754 (7)	154
O11—H11b⋯Br1^vii^	0.84	2.78	3.387 (6)	130
O12—H12a⋯Br10^viii^	0.84	2.84	3.599 (6)	152
O12—H12b⋯Br8^iii^	0.84	3.04	3.745 (4)	143
O13—H13a⋯O14^iv^	0.84	1.93	2.748 (6)	165
O13—H13b⋯Br5	0.84	2.83	3.463 (4)	134
O14—H14a⋯O10^iv^	0.84	1.97	2.749 (5)	153
O14—H14b⋯Br5	0.84	2.71	3.405 (4)	141
O15—H15a⋯Br12^ix^	0.84	2.82	3.531 (4)	143
O15—H15b⋯Br8^ix^	0.84	2.86	3.636 (6)	154

Symmetry codes: (i) 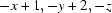; (ii) 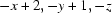; (iii) 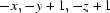; (iv) 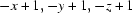; (v) 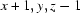; (vi) 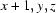; (vii) 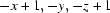; (viii) 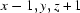; (ix) 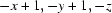.

**Fig. 1.**
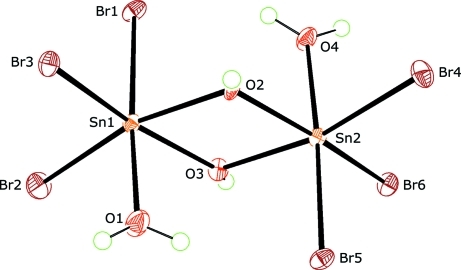
The molecular structure of the molecule occupying a general position in (I) showing displacement ellipsoids at the 50% probability level.

**Fig. 2.**
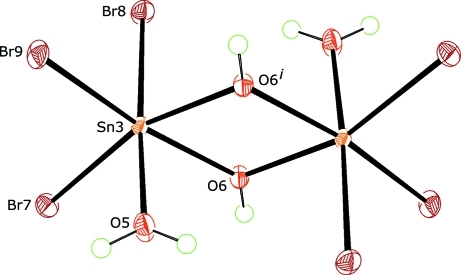
The molecular structure of the centrosymmetric molecule in (I) containing the Sn3 atom showing displacement ellipsoids at the 50% probability level. Symmetry operation i: 1-x, 2-y, -z.

**Fig. 3.**
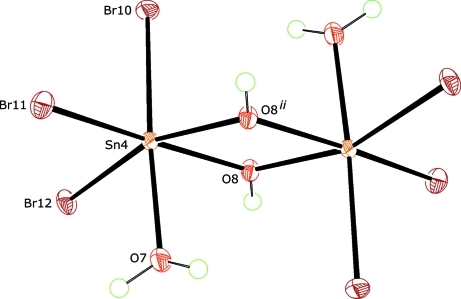
The molecular structure of the centrosymmetric molecule in (I) containing the Sn4 atom showing displacement ellipsoids at the 50% probability level. Symmetry operation ii: 2-x, 1-y, -z.

**Fig. 4.**
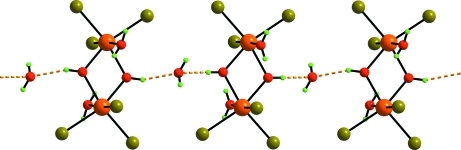
A view of a supramolecular chain in (I), aligned along [110], whereby the centrosymmetric dinuclear molecules, i.e. containing the Sn3 and Sn4 atoms, are bridged by Ohydroxyl–H···Owater hydrogen bonds (orange dashed lines). Colour code: Sn, orange; Br, olive; O, red; and H, green.

**Fig. 5.**
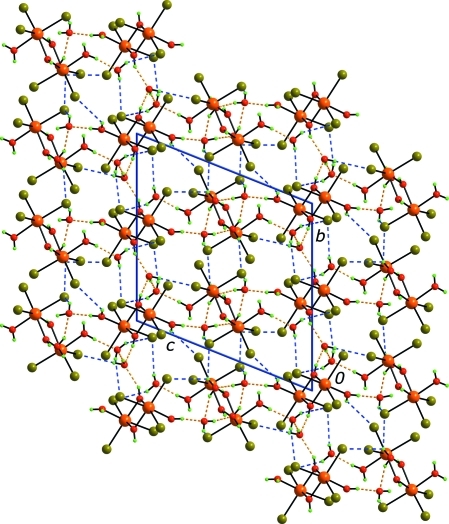
View in projection down the a axis of the unit cell contents in (I). The O–H···O and O–H···Br interactions are shown as orange and blue dashed lines, respectively. Colour code: Sn, orange; Br, olive; O, red; and H, green.

## supplementary crystallographic information

### Comment

The monoclinic (*P*2_1_/*c*) polymorph of the title compound was originally isolated as an hydrolysis product during recrystallisation experiments (Howie *et al.*, 2005). The present triclinic polymorph was isolated similarly as an hydrolysis product.

The crystallographic asymmetric unit of (I) comprises two formula units of [(H_2_O)Br_3_Sn(µ-OH)_2_SnBr_3_(OH_2_)] and seven water molecules of crystallisation. One of the [Br_3_(H_2_O)Sn(µ-OH)_2_SnBr_3_(OH_2_)] molecules occupies a general position, Fig. 1, whereas two are disposed about crystallographic centres of inversion, Figs 2 and 3. Nevertheless, the molecules are closely related in terms of overall geometry with differences relating primarily to variations in geometric parameters. Each dinuclear molecule features two Sn centres connected by symmetrically bridging hydroxyl groups, with two Br atoms lying in the plane of the central Sn_2_O_2_ core to form an equatorial Br_4_Sn_2_O_2_ framework. For each Sn atom, the third Br atom lies to one side of the plane and the coordinated water molecule to the other so that the water molecules are *anti*. The Sn–O_hydroxyl_ bond distances are systematically shorter than the Sn—O_aqua_ distances, and the Sn–Br_equatorial_ bond distances are shorter than the Sn—Br_axial_ bond distances. The elongation of the Sn—Br_axial_ bond distances partially relates to the participation of these atoms in intramolecular O_aqua_–H···Br hydrogen bonds which are systematically shorter than the intermolecular O_water_–H···Br hydrogen bonds, Table 1. The observed trends for the dinuclear species match those found in the monoclinic polymorph (Howie *et al.*, 2005) and other related di-µ-hydroxo-bis[*fac*-trichloroaquotin(IV)] complexes (Barnes *et al*, 1980; Cameron *et al.*, 1985; Shihada *et al.*, 2004; Müller *et al.*, 2007).

Extensive hydrogen bonding is found in the crystal structure that link all components into a 3-D network. Each hydroxyl group forms a single O_hydroxyl_–H···O_water_ hydrogen bond. In the case of the centrosymmetric molecules, *i*.*e*. containing the Sn3 and Sn4 atoms, single water molecules serve as bridges between them to form a supramolecular chain aligned along [1 1 0], Fig. 4 and Table 1. It is these O_hydroxyl_–H···O_water_ hydrogen bonds that appear to be the major difference between the the triclinic and monoclinic forms. In the monoclinic form, one hydroxyl group forms a single O_hydroxyl_–H···O_water_ hydrogen bond whereas the other forms two, with neither forming direct bridges between dinuclear molecules. In (I), each of the aqua molecules forms an intramolecular O_aqua_–H···Br hydrogen bond as well as an O_aqua_–H···O_water_ interaction, Table 1. Three of the lattice water molecules form two O_water_–H···Br hydrogen bonds and the remaining four water molecules form a single O_water_–H···Br hydrogen bond and an O_water_–H···O_water_ hydrogen bond. A view of the unit cell contents is shown in Fig. 5.

### Experimental

Solutions of PrS(═O)OCH_2_CH_2_S(═O)OPr (210 mg, 1 mmol) in MeOH (15 ml) and SnBr_4_ (440 mg, 1 mmol) in MeOH (15 ml) were mixed. After maintaining the reaction mixture at room temperature for several days, the microcrystalline precipitate was collected. As the crystals were not suitable for X-ray study, they were redissolved in MeOH and the solution was maintained at room temperature. After two weeks, colourless blocks of (I) suitable for X-ray analysis were collected and found to be hydrolysed stannic bromide. On heating the crystals, decomposition slowly occurred, and hence no melting point was measured. Standing in a moist atmosphere resulted in the formation of a syrup.

### Refinement

The O-bound H atoms were located from difference maps and refined with O–H = 0.840±0.001 Å, and with *U*_iso_(H) = 1.5*U_eq_*(C). The maximum and minimum residual electron density peaks of 1.06 and 1.63 e Å^-3^, respectively, were located 2.28 Å and 0.82 Å from the H6 and Sn2 atoms, respectively. The ADDSYM routine in PLATON (Spek, 2003) suggested the possibility of additional (C) symmetry. However, in this pseudo-symmetric setting, the O9-water molecule has no symmetry equivalent in the structure.

### Crystal data

**Table:**

[Sn_2_Br_6_(HO)_2_(H_2_O)_2_]_2_·7H_2_O	*Z* = 2
*M**_r_* = 1699.85	*F*(000) = 1532
Triclinic, *P*1	*D*_x_ = 3.304 Mg m^−^^3^
Hall symbol: -P 1	Mo *K*α radiation, λ = 0.71073 Å
*a* = 9.9652 (2) Å	Cell parameters from 22681 reflections
*b* = 14.0027 (3) Å	θ = 2.9–27.5°
*c* = 14.5230 (3) Å	µ = 16.97 mm^−^^1^
α = 64.8591 (13)°	*T* = 120 K
β = 69.9803 (13)°	Block, colourless
γ = 75.0492 (15)°	0.20 × 0.18 × 0.06 mm
*V* = 1708.54 (6) Å^3^	

### Data collection

**Table:**

Nonius KappaCCD diffractometer	7823 independent reflections
Radiation source: Enraf Nonius FR591 rotating anode	5613 reflections with *I* > 2σ(*I*)
10 cm confocal mirrors	*R*_int_ = 0.045
Detector resolution: 9.091 pixels mm^-1^	θ_max_ = 27.5°, θ_min_ = 3.0°
φ and ω scans	*h* = −12→12
Absorption correction: multi-scan (*SADABS*; Sheldrick, 2007)	*k* = −18→18
*T*_min_ = 0.421, *T*_max_ = 0.746	*l* = −18→18
36325 measured reflections	

### Refinement

**Table:**

Refinement on *F*^2^	Primary atom site location: structure-invariant direct methods
Least-squares matrix: full	Secondary atom site location: difference Fourier map
*R*[*F*^2^ > 2σ(*F*^2^)] = 0.035	Hydrogen site location: inferred from neighbouring sites
*wR*(*F*^2^) = 0.078	H-atom parameters constrained
*S* = 1.05	*w* = 1/[σ^2^(*F*_o_^2^) + (0.0218*P*)^2^ + 6.2493*P*] where *P* = (*F*_o_^2^ + 2*F*_c_^2^)/3
7823 reflections	(Δ/σ)_max_ = 0.001
352 parameters	Δρ_max_ = 1.06 e Å^−^^3^
36 restraints	Δρ_min_ = −1.63 e Å^−^^3^

### Special details

**Table:**

Geometry. All s.u.'s (except the s.u. in the dihedral angle between two l.s. planes) are estimated using the full covariance matrix. The cell s.u.'s are taken into account individually in the estimation of s.u.'s in distances, angles and torsion angles; correlations between s.u.'s in cell parameters are only used when they are defined by crystal symmetry. An approximate (isotropic) treatment of cell s.u.'s is used for estimating s.u.'s involving l.s. planes.
Refinement. Refinement of *F*^2^ against ALL reflections. The weighted *R*-factor wR and goodness of fit *S* are based on *F*^2^, conventional *R*-factors *R* are based on *F*, with *F* set to zero for negative *F*^2^. The threshold expression of *F*^2^ > 2σ(*F*^2^) is used only for calculating *R*-factors(gt) etc. and is not relevant to the choice of reflections for refinement. *R*-factors based on *F*^2^ are statistically about twice as large as those based on *F*, and *R*- factors based on ALL data will be even larger.

### Fractional atomic coordinates and isotropic or equivalent isotropic displacement parameters (Å^2^)

**Table:**

	*x*	*y*	*z*	*U*_iso_*/*U*_eq_	
Sn1	0.25914 (4)	0.18193 (3)	0.42146 (3)	0.01129 (9)	
Sn2	0.24278 (4)	0.32001 (3)	0.56732 (3)	0.01112 (9)	
Br1	0.14362 (6)	0.02944 (5)	0.58688 (4)	0.01635 (13)	
Br2	0.08773 (7)	0.19732 (5)	0.32194 (5)	0.02494 (15)	
Br3	0.47120 (6)	0.06473 (5)	0.35321 (4)	0.01606 (13)	
Br4	0.41823 (6)	0.30744 (5)	0.66344 (5)	0.02016 (14)	
Br5	0.35268 (6)	0.47436 (4)	0.40290 (4)	0.01513 (13)	
Br6	0.03158 (6)	0.43028 (5)	0.64553 (4)	0.01642 (13)	
O1	0.3524 (5)	0.3139 (3)	0.2907 (3)	0.0247 (10)	
H1A	0.4013	0.3141	0.2307	0.037*	
H1B	0.3687	0.3637	0.3015	0.037*	
O2	0.3683 (4)	0.2055 (3)	0.5078 (3)	0.0115 (8)	
H2	0.4587	0.1961	0.4879	0.017*	
O3	0.1324 (4)	0.2961 (3)	0.4820 (3)	0.0132 (8)	
H3	0.0442	0.3164	0.5023	0.020*	
O4	0.1573 (5)	0.1822 (3)	0.7003 (3)	0.0202 (9)	
H4A	0.1162	0.1900	0.7582	0.030*	
H4B	0.1506	0.1256	0.6964	0.030*	
Sn3	0.34919 (4)	0.94137 (3)	0.07239 (3)	0.01027 (9)	
Br7	0.32460 (6)	0.74736 (5)	0.17782 (4)	0.01670 (13)	
Br8	0.29454 (6)	0.94221 (5)	−0.08785 (4)	0.01846 (14)	
Br9	0.09746 (6)	1.02285 (5)	0.13907 (5)	0.01875 (14)	
O5	0.4029 (4)	0.9463 (4)	0.2010 (3)	0.0196 (9)	
H5A	0.3490	0.9284	0.2628	0.029*	
H5B	0.4623	0.9824	0.1963	0.029*	
O6	0.5706 (4)	0.9129 (3)	0.0104 (3)	0.0134 (8)	
H6	0.6306	0.8575	0.0222	0.020*	
Sn4	0.84937 (4)	0.44058 (3)	0.06715 (3)	0.01050 (9)	
Br10	0.80194 (6)	0.44339 (5)	−0.09571 (4)	0.01709 (13)	
Br11	0.81979 (6)	0.24771 (5)	0.17183 (4)	0.01730 (13)	
Br12	0.59787 (6)	0.52341 (5)	0.13434 (4)	0.01666 (13)	
O7	0.8931 (4)	0.4524 (4)	0.1967 (3)	0.0187 (9)	
H7A	0.8399	0.4282	0.2583	0.028*	
H7B	0.9802	0.4440	0.1955	0.028*	
O8	1.0732 (4)	0.4123 (3)	0.0172 (3)	0.0129 (8)	
H8	1.1140	0.3541	0.0108	0.019*	
O9	0.2294 (4)	0.2630 (4)	0.9291 (3)	0.0220 (10)	
H9A	0.1629	0.2430	0.9213	0.033*	
H9B	0.2910	0.2744	0.8704	0.033*	
O10	0.7781 (3)	0.10636 (17)	0.61362 (8)	0.0187 (9)	
H10A	0.8390	0.0516	0.6174	0.028*	
H10B	0.7909	0.1420	0.5487	0.028*	
O11	0.6517 (4)	0.1932 (4)	0.4475 (3)	0.0203 (9)	
H11A	0.6948	0.2473	0.4162	0.030*	
H11B	0.6769	0.1611	0.4056	0.030*	
O12	0.0169 (5)	0.1982 (4)	0.8889 (3)	0.0230 (10)	
H12A	−0.0473	0.2509	0.8786	0.034*	
H12B	−0.0164	0.1438	0.9380	0.034*	
O13	0.7169 (4)	0.3949 (3)	0.3846 (3)	0.0198 (9)	
H13A	0.7430	0.3733	0.4401	0.030*	
H13B	0.6518	0.4472	0.3810	0.030*	
O14	0.1530 (4)	0.6926 (3)	0.4532 (3)	0.0198 (9)	
H14A	0.1844	0.7510	0.4139	0.030*	
H14B	0.1763	0.6534	0.4181	0.030*	
O15	0.4686 (5)	0.2973 (4)	0.1094 (3)	0.0310 (12)	
H15A	0.4739	0.3565	0.0591	0.047*	
H15B	0.5446	0.2560	0.0981	0.047*	

### Atomic displacement parameters (Å^2^)

**Table:**

	*U*^11^	*U*^22^	*U*^33^	*U*^12^	*U*^13^	*U*^23^
Sn1	0.01050 (19)	0.0111 (2)	0.01327 (19)	−0.00171 (15)	−0.00335 (15)	−0.00510 (15)
Sn2	0.01002 (19)	0.0115 (2)	0.01214 (19)	−0.00217 (15)	−0.00210 (15)	−0.00487 (15)
Br1	0.0138 (3)	0.0120 (3)	0.0189 (3)	−0.0042 (2)	0.0015 (2)	−0.0049 (2)
Br2	0.0254 (3)	0.0261 (4)	0.0320 (4)	−0.0023 (3)	−0.0183 (3)	−0.0111 (3)
Br3	0.0142 (3)	0.0179 (3)	0.0168 (3)	−0.0013 (2)	−0.0012 (2)	−0.0098 (2)
Br4	0.0177 (3)	0.0263 (4)	0.0216 (3)	−0.0024 (2)	−0.0099 (2)	−0.0104 (3)
Br5	0.0135 (3)	0.0120 (3)	0.0165 (3)	−0.0038 (2)	0.0001 (2)	−0.0042 (2)
Br6	0.0136 (3)	0.0178 (3)	0.0174 (3)	−0.0010 (2)	−0.0004 (2)	−0.0097 (2)
O1	0.036 (3)	0.018 (3)	0.017 (2)	−0.011 (2)	0.0016 (19)	−0.0073 (19)
O2	0.0089 (19)	0.011 (2)	0.018 (2)	0.0024 (16)	−0.0036 (16)	−0.0103 (16)
O3	0.0056 (18)	0.017 (2)	0.019 (2)	0.0017 (16)	−0.0038 (16)	−0.0095 (17)
O4	0.032 (3)	0.013 (2)	0.014 (2)	−0.0097 (19)	0.0004 (18)	−0.0052 (18)
Sn3	0.00835 (19)	0.0102 (2)	0.01140 (19)	−0.00138 (14)	−0.00144 (14)	−0.00403 (15)
Br7	0.0183 (3)	0.0110 (3)	0.0193 (3)	−0.0030 (2)	−0.0053 (2)	−0.0034 (2)
Br8	0.0174 (3)	0.0256 (3)	0.0156 (3)	−0.0059 (2)	−0.0051 (2)	−0.0084 (2)
Br9	0.0103 (3)	0.0184 (3)	0.0210 (3)	0.0006 (2)	0.0016 (2)	−0.0071 (2)
O5	0.020 (2)	0.028 (3)	0.013 (2)	−0.0127 (19)	−0.0002 (17)	−0.0071 (18)
O6	0.010 (2)	0.008 (2)	0.018 (2)	0.0007 (15)	−0.0015 (16)	−0.0038 (16)
Sn4	0.00841 (19)	0.0117 (2)	0.01130 (19)	−0.00140 (14)	−0.00110 (14)	−0.00534 (15)
Br10	0.0162 (3)	0.0242 (3)	0.0150 (3)	−0.0041 (2)	−0.0046 (2)	−0.0099 (2)
Br11	0.0181 (3)	0.0122 (3)	0.0200 (3)	−0.0029 (2)	−0.0054 (2)	−0.0037 (2)
Br12	0.0104 (3)	0.0172 (3)	0.0183 (3)	−0.0006 (2)	0.0012 (2)	−0.0074 (2)
O7	0.014 (2)	0.029 (3)	0.015 (2)	−0.0080 (19)	−0.0008 (17)	−0.0085 (19)
O8	0.0090 (19)	0.010 (2)	0.018 (2)	−0.0004 (16)	−0.0002 (16)	−0.0070 (17)
O9	0.019 (2)	0.022 (2)	0.021 (2)	−0.0041 (19)	−0.0007 (18)	−0.0075 (19)
O10	0.017 (2)	0.019 (2)	0.014 (2)	−0.0012 (17)	−0.0005 (16)	−0.0043 (17)
O11	0.016 (2)	0.032 (3)	0.017 (2)	−0.0057 (19)	−0.0008 (17)	−0.0138 (19)
O12	0.022 (2)	0.022 (3)	0.018 (2)	−0.0051 (19)	−0.0030 (18)	−0.0018 (19)
O13	0.016 (2)	0.020 (3)	0.019 (2)	−0.0022 (17)	−0.0028 (18)	−0.0050 (19)
O14	0.018 (2)	0.021 (3)	0.025 (2)	−0.0048 (19)	−0.0010 (18)	−0.0152 (19)
O15	0.038 (3)	0.024 (3)	0.019 (2)	0.002 (2)	−0.001 (2)	−0.006 (2)

### Geometric parameters (Å, °)

**Table:**

Sn1—O3	2.078 (4)	O6—Sn3^i^	2.086 (4)
Sn1—O2	2.082 (4)	O6—H6	0.840
Sn1—O1	2.140 (4)	Sn4—O8	2.083 (4)
Sn1—Br2	2.5078 (7)	Sn4—O8^ii^	2.090 (4)
Sn1—Br3	2.5100 (7)	Sn4—O7	2.150 (4)
Sn1—Br1	2.5830 (7)	Sn4—Br11	2.5114 (7)
Sn2—O2	2.070 (4)	Sn4—Br12	2.5230 (6)
Sn2—O3	2.082 (4)	Sn4—Br10	2.5487 (7)
Sn2—O4	2.176 (4)	O7—H7A	0.840
Sn2—Br6	2.5062 (7)	O7—H7B	0.840
Sn2—Br4	2.5180 (7)	O8—Sn4^ii^	2.090 (4)
Sn2—Br5	2.5726 (7)	O8—H8	0.840
O1—H1A	0.840	O9—H9A	0.840
O1—H1B	0.840	O9—H9B	0.840
O2—H2	0.840	O10—H10A	0.840
O3—H3	0.840	O10—H10B	0.840
O4—H4A	0.840	O11—H11A	0.840
O4—H4B	0.840	O11—H11B	0.840
Sn3—O6	2.080 (4)	O12—H12A	0.840
Sn3—O6^i^	2.086 (4)	O12—H12B	0.840
Sn3—O5	2.144 (4)	O13—H13A	0.840
Sn3—Br9	2.5161 (6)	O13—H13B	0.840
Sn3—Br7	2.5173 (7)	O14—H14A	0.840
Sn3—Br8	2.5599 (7)	O14—H14B	0.840
O5—H5A	0.840	O15—H15A	0.840
O5—H5B	0.840	O15—H15B	0.840
			
O3—Sn1—O2	71.72 (14)	O6^i^—Sn3—Br9	94.07 (10)
O3—Sn1—O1	84.91 (16)	O5—Sn3—Br9	88.25 (12)
O2—Sn1—O1	86.80 (17)	O6—Sn3—Br7	93.77 (11)
O3—Sn1—Br2	93.67 (11)	O6^i^—Sn3—Br7	163.58 (11)
O2—Sn1—Br2	165.00 (10)	O5—Sn3—Br7	88.16 (12)
O1—Sn1—Br2	88.57 (13)	Br9—Sn3—Br7	99.89 (2)
O3—Sn1—Br3	162.73 (11)	O6—Sn3—Br8	92.68 (11)
O2—Sn1—Br3	92.64 (10)	O6^i^—Sn3—Br8	93.59 (11)
O1—Sn1—Br3	86.98 (12)	O5—Sn3—Br8	177.00 (11)
Br2—Sn1—Br3	101.35 (2)	Br9—Sn3—Br8	93.09 (2)
O3—Sn1—Br1	91.90 (11)	Br7—Sn3—Br8	94.25 (2)
O2—Sn1—Br1	90.45 (11)	Sn3—O5—H5A	123
O1—Sn1—Br1	176.32 (12)	Sn3—O5—H5B	126
Br2—Sn1—Br1	93.48 (2)	H5A—O5—H5B	108
Br3—Sn1—Br1	95.61 (2)	Sn3—O6—Sn3^i^	108.54 (17)
O2—Sn2—O3	71.86 (14)	Sn3—O6—H6	133
O2—Sn2—O4	82.88 (15)	Sn3^i^—O6—H6	117
O3—Sn2—O4	87.66 (16)	O8—Sn4—O8^ii^	72.01 (17)
O2—Sn2—Br6	162.69 (11)	O8—Sn4—O7	82.89 (15)
O3—Sn2—Br6	94.12 (10)	O8^ii^—Sn4—O7	83.46 (16)
O4—Sn2—Br6	86.53 (11)	O8—Sn4—Br11	94.42 (11)
O2—Sn2—Br4	93.57 (11)	O8^ii^—Sn4—Br11	165.70 (11)
O3—Sn2—Br4	165.24 (11)	O7—Sn4—Br11	90.50 (12)
O4—Sn2—Br4	88.16 (12)	O8—Sn4—Br12	161.64 (11)
Br6—Sn2—Br4	99.75 (2)	O8^ii^—Sn4—Br12	93.07 (10)
O2—Sn2—Br5	93.23 (11)	O7—Sn4—Br12	84.86 (11)
O3—Sn2—Br5	90.43 (11)	Br11—Sn4—Br12	99.31 (2)
O4—Sn2—Br5	176.04 (11)	O8—Sn4—Br10	96.35 (11)
Br6—Sn2—Br5	97.08 (2)	O8^ii^—Sn4—Br10	91.56 (11)
Br4—Sn2—Br5	92.83 (2)	O7—Sn4—Br10	174.96 (12)
Sn1—O1—H1A	126	Br11—Sn4—Br10	94.52 (2)
Sn1—O1—H1B	120	Br12—Sn4—Br10	94.66 (2)
H1A—O1—H1B	111	Sn4—O7—H7A	120
Sn2—O2—Sn1	108.35 (16)	Sn4—O7—H7B	117
Sn2—O2—H2	126	H7A—O7—H7B	112
Sn1—O2—H2	117	Sn4—O8—Sn4^ii^	107.99 (17)
Sn1—O3—Sn2	108.06 (16)	Sn4—O8—H8	120
Sn1—O3—H3	137	Sn4^ii^—O8—H8	127
Sn2—O3—H3	109	H9A—O9—H9B	103
Sn2—O4—H4A	117	H10A—O10—H10B	105
Sn2—O4—H4B	125	H11A—O11—H11B	106
H4A—O4—H4B	117	H12A—O12—H12B	112
O6—Sn3—O6^i^	71.46 (17)	H13A—O13—H13B	111
O6—Sn3—O5	85.38 (16)	H14A—O14—H14B	109
O6^i^—Sn3—O5	83.63 (16)	H15A—O15—H15B	110
O6—Sn3—Br9	164.73 (11)		
			
O3—Sn2—O2—Sn1	0.38 (16)	O2—Sn2—O3—Sn1	−0.38 (16)
O4—Sn2—O2—Sn1	90.24 (19)	O4—Sn2—O3—Sn1	−83.65 (18)
Br6—Sn2—O2—Sn1	37.5 (5)	Br6—Sn2—O3—Sn1	−169.99 (14)
Br4—Sn2—O2—Sn1	177.94 (14)	Br4—Sn2—O3—Sn1	−10.0 (6)
Br5—Sn2—O2—Sn1	−89.02 (15)	Br5—Sn2—O3—Sn1	92.88 (15)
O3—Sn1—O2—Sn2	−0.38 (16)	O6^i^—Sn3—O6—Sn3^i^	0.0
O1—Sn1—O2—Sn2	85.32 (19)	O5—Sn3—O6—Sn3^i^	−84.82 (19)
Br2—Sn1—O2—Sn2	13.1 (5)	Br9—Sn3—O6—Sn3^i^	−19.2 (5)
Br3—Sn1—O2—Sn2	172.14 (14)	Br7—Sn3—O6—Sn3^i^	−172.66 (15)
Br1—Sn1—O2—Sn2	−92.23 (15)	Br8—Sn3—O6—Sn3^i^	92.89 (15)
O2—Sn1—O3—Sn2	0.37 (16)	O8^ii^—Sn4—O8—Sn4^ii^	0.0
O1—Sn1—O3—Sn2	−87.9 (2)	O7—Sn4—O8—Sn4^ii^	−85.40 (19)
Br2—Sn1—O3—Sn2	−176.16 (14)	Br11—Sn4—O8—Sn4^ii^	−175.36 (14)
Br3—Sn1—O3—Sn2	−25.6 (5)	Br12—Sn4—O8—Sn4^ii^	−36.9 (4)
Br1—Sn1—O3—Sn2	90.23 (15)	Br10—Sn4—O8—Sn4^ii^	89.59 (15)

Symmetry codes: (i) −*x*+1, −*y*+2, −*z*; (ii) −*x*+2, −*y*+1, −*z*.

### Hydrogen-bond geometry (Å, °)

**Table:**

*D*—H···*A*	*D*—H	H···*A*	*D*···*A*	*D*—H···*A*
O1—H1a···O15	0.84	1.75	2.573 (6)	165
O1—H1b···Br5	0.84	2.50	3.290 (4)	157
O2—H2···O11	0.84	1.80	2.638 (6)	172
O3—H3···O14^iii^	0.84	1.87	2.657 (6)	156
O4—H4a···O12	0.84	1.85	2.692 (6)	175
O4—H4b···Br1	0.84	2.51	3.257 (4)	149
O5—H5a···O10^iv^	0.84	1.76	2.592 (4)	174
O5—H5b···Br8^i^	0.84	2.60	3.329 (5)	146
O6—H6···O9^iv^	0.84	1.93	2.764 (7)	169
O7—H7a···O13	0.84	1.76	2.600 (6)	172
O7—H7b···Br10^ii^	0.84	2.64	3.307 (5)	137
O8—H8···O9^v^	0.84	1.99	2.768 (7)	154
O9—H9a···O12	0.84	1.98	2.817 (7)	175
O9—H9b···Br4	0.84	2.71	3.522 (4)	162
O10—H10a···Br1^vi^	0.84	2.87	3.456 (3)	129
O10—H10b···O11	0.84	2.14	2.772 (5)	132
O11—H11a···O13	0.84	1.97	2.754 (7)	154
O11—H11b···Br1^vii^	0.84	2.78	3.387 (6)	130
O12—H12a···Br10^viii^	0.84	2.84	3.599 (6)	152
O12—H12b···Br8^iii^	0.84	3.04	3.745 (4)	143
O13—H13a···O14^iv^	0.84	1.93	2.748 (6)	165
O13—H13b···Br5	0.84	2.83	3.463 (4)	134
O14—H14a···O10^iv^	0.84	1.97	2.749 (5)	153
O14—H14b···Br5	0.84	2.71	3.405 (4)	141
O15—H15a···Br12^ix^	0.84	2.82	3.531 (4)	143
O15—H15b···Br8^ix^	0.84	2.86	3.636 (6)	154

Symmetry codes:  (iii) −*x*, −*y*+1, −*z*+1; (iv) −*x*+1, −*y*+1, −*z*+1; (i) −*x*+1, −*y*+2, −*z*; (ii) −*x*+2, −*y*+1, −*z*; (v) *x*+1, *y*, *z*−1; (vi) *x*+1, *y*, *z*; (vii) −*x*+1, −*y*, −*z*+1; (viii) *x*−1, *y*, *z*+1; (ix) −*x*+1, −*y*+1, −*z*.
